# Triglyceride-glucose index linked to all-cause mortality in critically ill patients: a cohort of 3026 patients

**DOI:** 10.1186/s12933-022-01563-z

**Published:** 2022-07-08

**Authors:** Ying Liao, Rongting Zhang, Shanshan Shi, Yukun Zhao, Yibo He, Lihua Liao, Xueqin Lin, Qian Guo, Yani Wang, Liling Chen, Weiguo Li, Shihai Li, Kaihong Chen, Yong Fang

**Affiliations:** 1 Department of Cardiology, Longyan First Affiliated Hospital of Fujian Medical University, Longyan, 364000 China; 2 The Graduate School of Clinical Medicine, Fujian Medical University, Fuzhou, 350000 China; 3 Department of Cardiology, Guangdong Cardiovascular Institute, Guangdong Provincial People’s Hospital, Guangdong Academy of Medical Sciences, Guangzhou, 510080 China; 4 Department of Guangdong Provincial Key Laboratory of Coronary Heart Disease Prevention, Guangdong Cardiovascular Institute, Guangdong Provincial People’s Hospital, Guangdong Academy of Medical Sciences, Guangzhou, 510080 China; 5 Department of Anesthesiology, Longyan First Affiliated Hospital of Fujian Medical University, Longyan, 364000 China

**Keywords:** Triglyceride-glucose index, Insulin resistance, Intensive care unit, All-cause mortality

## Abstract

**Background:**

Triglyceride-glucose (TyG) index as a reliable surrogate of insulin resistance (IR) has been shown to be related to adverse clinical outcomes in patients with acute coronary syndrome, heart failure, ischemic stroke and so on. However, the relationship between TyG index and all-cause mortality in intensive care unit (ICU) patients remains unknown. The purpose of this study was to investigate the correlation between TyG index and all-cause mortality to evaluate the impact of IR on the prognosis of this population.

**Methods:**

This was a retrospective observational study that included 3026 patients who had an initial triglyceride and glucose data on the first day of ICU admission, and all data were extracted from the Medical Information Mart for Intensive Care III (MIMIC-III) database. These patients were grouped into quartiles (Q1–Q4) according to TyG index. The Kaplan–Meier analysis was used to compare all-cause mortality among the above four groups. Cox proportional hazards analyses were performed to examine the association between TyG index and all-cause mortality.

**Results:**

During 10.46 years of follow-up, 1148 (37.9%) patients died, of which 350 (11.6%) occurred during the hospital stay and 258 (8.5%) occurred during the ICU stay. Kaplan–Meier analysis showed that the risk of all-cause mortality was significantly higher in patients with higher TyG index (log-rank *P* = 0.021). Multivariable Cox proportional hazards analyses showed that the TyG index was an independent risk predictor of ICU death (HR: 1.72, 95% CI 1.18–2.52, *P* = 0.005) and hospital death (HR: 2.19, 95% CI 1.59–3.03, *P* < 0.001), and each 1-unit increased in the TyG index, a 1.19-fold increase in the risk of death during the hospital stay.

**Conclusions:**

TyG index is strongly related to the all-cause mortality increasing in critically ill patients. This finding indicates that the TyG index might be useful in identifying people at high risk of ICU death and hospital death.

**Supplementary Information:**

The online version contains supplementary material available at 10.1186/s12933-022-01563-z.

## Introduction

Patients in intensive care unit (ICU) usually have a longer duration of mean stay and extremely high mortality rates, placing a heavy burden to the family and society [1]. Risk prediction in advance is important to guide medical treatment. Severity of illness scores have gained considerable interest for their use in predicting outcomes such as mortality and length of stay [2–4]. However, these scores are far from perfect to predict the outcomes of critical illness [5]. Furthermore, these scores encompass various clinical information including patients’ symptoms, signs, laboratory tests, microbiology findings. In the absence of any of this information, these scores cannot be used [4]. Therefore, we urgently need to explore novel biomarkers, mechanisms, and targeted measures in ICU patients. Compared with these scores, the triglyceride-glucose (TyG) index is an easily available, inexpensive and reliable test, which may facilitate its use in the clinical practice [6].

Insulin resistance (IR), defined as the decreased insulin sensitivity of peripheral tissues, plays a critical role in many metabolic abnormalities associated with critical illness [7, 8], and is linked to increased morbidity and mortality [9]. Previous studies have found that critically ill patients had a 50% to 70% of reduction in insulin sensitivity compared to healthy controls [10–12]. Zauner et al. [12] demonstrated that the critically ill patients occurred severe IR on the first day after admission to ICU, and IR was related to the severity of their condition rather than the different admission diagnoses of ICU patients. IR, with time, contributes to micro- and macroangiopathy, neuropathy, and organ failure [13]. Accordingly, it is critical to identify early IR in critically ill patients.

TyG index based on fasting blood glucose (FBG) and triglycerides has been used in clinical practice as a simple and reliable surrogate marker of IR, and former studies have proved that it has high correlation with hyperinsulinaemic–euglycaemic clamp (the gold standard technique for assessing IR) [14, 15]. Previous studies have noted that the TyG index was related to adverse clinical outcomes in patients with acute coronary syndrome (ACS), heart failure (HF), ischemic stroke and so on [16–18]. However, current data about associations between TyG index and critically ill patients are limited, whether TyG index was an independent factor of prognosis in ICU patients has not been determined yet. The purpose of this study was to investigate the relationship between TyG index and all-cause mortality to evaluate the impact of IR on the prognosis of ICU patients.

## Methods

### Study population

This study is a retrospective observational study. Data were extracted from an online international database, the Medical Information Mart for Intensive Care III (MIMIC-III) [19]. The MIMIC-III is a longitudinal, single-center database which is maintained by the Laboratory of Computational Physiology at Massachusetts Institute of Technology (MIT). This database comprises information related to patients admitted to critical care units at a large tertiary care hospital located in Boston between June 1, 2001 and October 10, 2012. Access to this database is granted by passing an examination and obtaining certification. One author (YL) obtained permission to access the dataset (Record ID 36132841) and was responsible for data extraction. The project was approved by the institutional review boards of the MIT and Beth Israel Deaconess Medical Center (BIDMC) and was granted a waiver of informed consent.

**Fig. 1:**
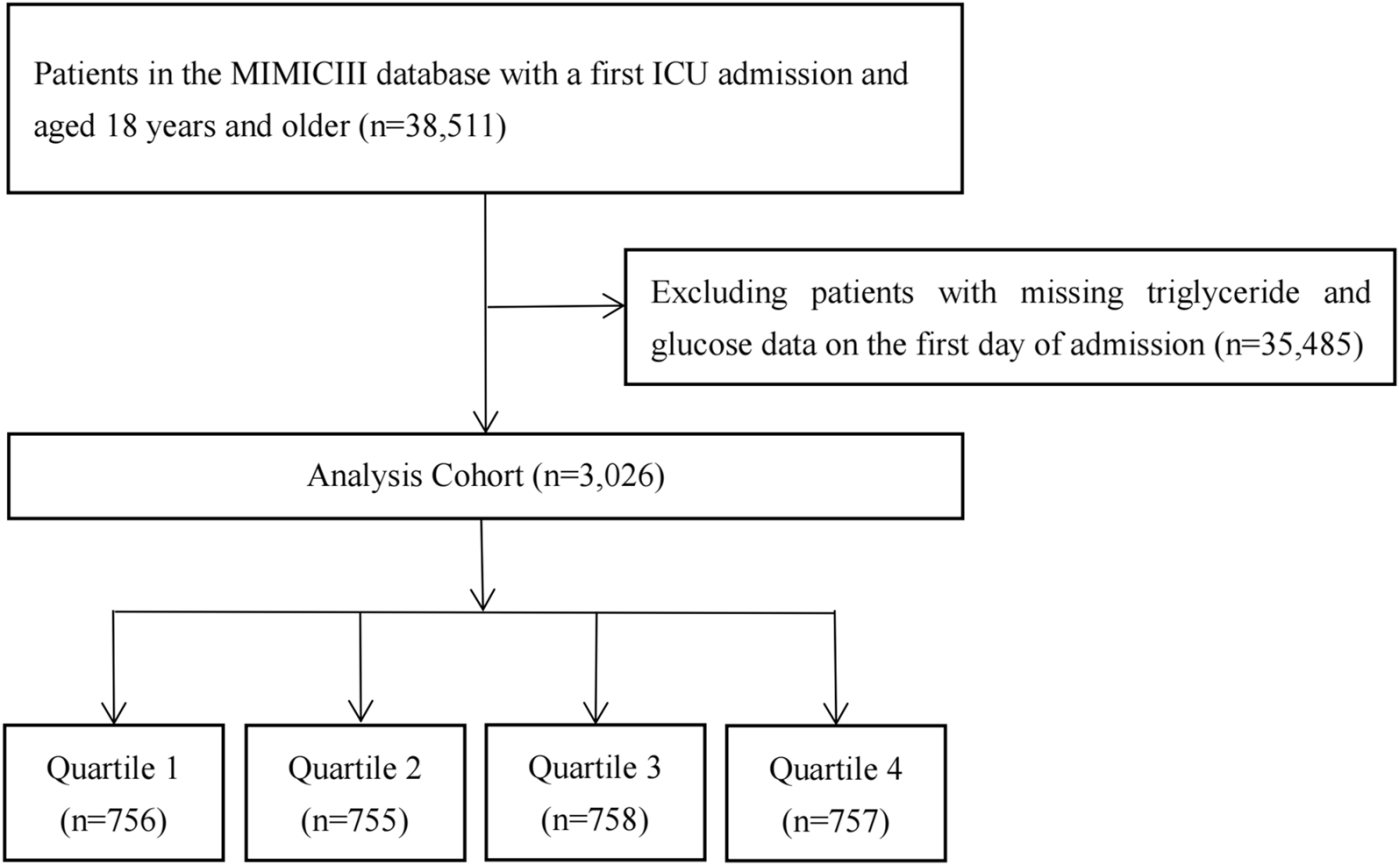
Flow chart of patients selection for analytic

We included 38,511 patients (aged ≥ 18 years) admitted to the ICU in MIMIC-III, and patients with missing triglyceride and glucose data on the first day of admission were excluded. Additionally, we analyzed only the first ICU stay for patients who were admitted to the ICU more than once. A total of 3026 patients were included in the final study cohort and divided into four groups based on the quartiles of the first day of ICU stay TyG index. Patient screening flow chart is displayed in Fig. 1.

### Variable extraction

Data on baseline characteristics within first 24 h of ICU admission were extracted from the MIMIC-III database, including sex, age, ethnicity, height, weight, body mass index, first care unit, severity at admission measured by Sequential Organ Failure Assessment (SOFA) score, Logistic Organ Dysfunction System (LODS) score, Systemic inflammatory response syndrome (SIRS) score, Oxford Acute Severity of Illness Score (OASIS), Acute physiology score III (APSIII), Simplified acute physiological score II (SAPSII). Initial TyG index [20] and vital signs were also extracted. The TyG index was calculated as the ln [Fasting triglyceride (mg/dL) × fasting glucose (mg/dL)]/2. If a variable was recorded more than once in the first 24 h, we used its average value. Comorbidities identified based on documented ICD-9 codes included coronary heart disease (CHD), HF, hypertension, atrial fibrillation (AF), dyslipidemia, diabetes mellitus (DM), chronic obstructive pulmonary disease (COPD), renal failure (RF), liver disease (LD), chronic kidney disease (CKD), sepsis, cancer. Acute kidney injury (AKI) was defined according to Kidney Disease: Improving Global Outcomes (KDIGO) guidelines as an increase in serum creatinine (Scr) by ≥ 0.3 mg/dL from baseline within 48 h [21]. Routine blood, complete set of biochemistry and laboratory indicators related to glucose and lipid metabolism were collected for analysis.

### Primary outcome and secondary outcomes

The primary outcome of this study was in hospital all-cause mortality. Of these, hospital death included ICU death and general ward inpatient death. The secondary outcomes included long-term follow-up death and length of stay in ICU and hospital. Patient mortality information for discharged patients was accessed from the US Social Security Death Index.

### Statistical analysis

The study population was divided into four groups based on the quartiles of the first day of ICU stay TyG index. Data were presented as the mean with standard deviation (SD) or median with interquartile range (IQR) for continuous variables and quantity and frequency (%) for categorical variables. Continuous variables were compared by Student’s t test and categorical variables were compared using the Pearson chi-square test or Fisher’s exact test. The propensity score matching (PSM) was used to adjust for covariates to ensure the comparability across groups in the analysis of baseline characteristics. Baseline characteristics of the original and matched cohorts were presented separately.

After testing the normality of the TyG index, multifactorial linear regression was used to analyze the relationship between TyG index with length of ICU stay and length of hospital stay. To evaluate the association between the primary outcomes and TyG index (1 unit and quartile), we used Kaplan Meier survival analysis to evaluate the incidence rate of primary outcome events among groups according to different levels of the TyG index, and discrepancies among groups were evaluated by log-rank test. We used Cox proportional hazards models to estimate the hazard ratio (HR) and 95% confidence interval (CI) between the risk of TyG index and primary outcomes. Moreover, the restricted cubic spline (RCS) regression model with assumed three knots was used to outline the relations between TyG index and HR. Further stratified analyses according to sex, age (≤ 65 and > 65 years), body mass index (BMI) (< 30 and ≥ 30 kg/m^2^), DM and hypertension were conducted to identify the consistency of the prognostic impact of TyG index for primary endpoint. The interaction between TyG index and stratified variables was further tested. The baseline variables were used as candidate predictors for the multivariate regression model. Considering the possibility of overfitting, we quantified the multicollinearity between variables using variance inflation factors (VIF). Variables with VIF ≥ 5 were excluded. Finally, the model was adjusted for age, sex, ethnicity, first care unit, SOFA score, LODS score, laboratory tests [white blood cell (WBC), red blood cell (RBC), hemoglobin, serum sodium, serum potassium, total cholesterol (TC), low-density lipoprotein (LDL), high-density lipoprotein (HDL), albumin, Scr], and co-morbidities [CHD, HF, hypertension, dyslipidemia, DM, COPD, RF, LD, CKD, AKI, sepsis, cancer].

To assess whether the accuracy of predicting adverse outcome events would be improved by adding TyG index to the existing severity of illness scores (SOFA score, LODS score, OASIS score, SIRS score, APSIII, SAPSII), the area under the curve (AUC) was calculated. The results of AUC were generated from the matched cohort.

All data analyses were performed using R software (version 4.0.4; R Foundation for Statistical Computing, Vienna, Austria) and SPSS statistical software (IBM SPSS Statistics, Version 24.0; Armonk, NY, USA). A two-sided *P*-value < 0.05 was considered statistically significant for all analyses.

## Results

After reviewing the data of 38,511 patients who were admitted into the ICU from the MIMIC-III database, a total of 3026 were included in our study. The mean age of the enrolled patients was 65.44 ± 16.07 years, including 1,240 (41%) female patients. All enrolled patients’ average TyG index was 9.16 ± 0.74. During 10.46 years of follow-up, 1148 (37.9%) experienced all-cause mortality, of which 350 (11.6%) occurred during the hospital stay and 258 (8.5%) occurred during the ICU stay.

### Baseline characteristics

Baseline characteristics grouped according to quartiles of the TyG index are shown in Table 1. Patients with higher TyG index were generally younger, higher severity of illness scores on admission, higher prevalence of DM, COPD, RF, LD, AKI, CKD, sepsis, higher levels of WBC, Scr, TC, LDL, hemoglobin A1c and blood urea nitrogen, lower levels of albumin and HDL compared to the lower group. With increasing TyG index, ICU length of stay (3.68 days vs. 3.78 days vs. 4.16 days vs. 5.49 days, *P* < 0.001), hospital length of stay (7.68 days vs. 7.83 days vs. 8.82 days vs. 10.50 days, *P* < 0.001), ICU mortality (5.6% vs. 7.8% vs. 10.2% vs. 10.6%, *P* = 0.001), hospital mortality (7.5% vs. 11.3% vs. 13.5% vs. 14.0%, *P* < 0.001), and long-term follow-up mortality (35.1% vs. 35.8% vs. 40.4% vs. 40.6%, *P* = 0.040) increased gradually. Similar results were observed for the baseline characteristics grouped by the TyG index and baseline characteristics of the matched cohorts (Additional file 1: Table S1).

**Table 1:** Baseline characteristics of ICU patients grouped according to TyG index quartiles

Categories	Overall (N = 3026)	Q1 (N = 756)	Q2 (N = 755)	Q3 (N = 758)	Q4 (N = 757)	*P*-value
Demographic						
Age, years, mean (SD)	65.44 (16.07)	68.32 (16.44)	66.83 (15.90)	65.62 (15.58)	60.99 (15.45)	< 0.001
Male, n (%)	1786 (59.0)	451 (59.7)	437 (57.9)	455 (60.0)	443 (58.5)	0.819
Ethnicity, n (%)						0.003
Asian	617 (20.4)	135 (17.9)	139 (18.4)	170 (22.4)	173 (22.9)	
Black	58 (1.9)	21 (2.8)	10 (1.3)	19 (2.5)	8 (1.1)	
White	202 (6.7)	62 (8.2)	44 (5.8)	36 (4.7)	60 (7.9)	
Hispanic/Latino	83 (2.7)	15 (2.0)	25 (3.3)	21 (2.8)	22 (2.9)	
Other	2066 (68.3)	523 (69.2)	537 (71.1)	512 (67.5)	494 (65.3)	
Weight, kg, mean (SD)	82.25 (22.71)	77.00 (19.72)	80.49 (22.39)	82.28 (21.75)	88.98 (24.93)	< 0.001
Height, cm, mean (SD)	169.92 (14.47)	171.03 (20.16)	169.30 (13.36)	169.27 (12.04)	170.14 (11.20)	0.319
BMI, kg/m^2^, mean (SD)	31.35 (76.35)	36.20 (156.02)	29.21 (12.59)	29.18 (13.20)	31.10 (12.03)	0.581
ICU admission						
SOFA score, mean (SD)	3.32 (2.91)	2.86 (2.45)	3.00 (2.59)	3.42 (2.88)	4.01 (3.48)	< 0.001
LODS score, mean (SD)	3.49 (2.56)	3.08 (2.25)	3.20 (2.26)	3.64 (2.61)	4.05 (2.94)	< 0.001
OASIS score, mean (SD)	30.94 (8.96)	29.81 (8.58)	30.45 (8.38)	31.29 (9.04)	32.22 (9.61)	< 0.001
SIRS score, mean (SD)	2.51 (1.06)	2.32 (1.04)	2.40 (1.06)	2.57 (1.03)	2.73 (1.05)	< 0.001
APSIII, mean (SD)	39.29 (18.74)	36.61 (16.12)	37.10 (17.36)	39.37 (19.18)	44.05 (21.01)	< 0.001
SAPSII, mean (SD)	32.83 (13.23)	31.50 (12.09)	32.08 (12.33)	33.39 (13.59)	34.37 (14.58)	< 0.001
First Care Unit, n (%)						< 0.001
CCU	1259 (41.6)	332 (43.9)	304 (40.3)	333 (43.9)	290 (38.3)	
CSRU	152 (5.0)	27 (3.6)	36 (4.8)	45 (5.9)	44 (5.8)	
MICU	708 (23.4)	152 (20.1)	162 (21.5)	159 (21.0)	235 (31.0)	
SICU	643 (21.2)	180 (23.8)	176 (23.3)	154 (20.3)	133 (17.6)	
TSICU	264 (8.7)	65 (8.6)	77 (10.2)	67 (8.8)	55 (7.3)	
Vital signs						
HR, bmp, mean (SD)	81.13 (16.20)	78.03 (15.21)	79.34 (15.65)	82.02 (15.79)	85.15 (17.18)	< 0.001
SBP, mmHg, mean (SD)	123.12 (18.63)	122.37 (18.77)	123.36 (18.51)	123.58 (19.21)	123.17 (18.03)	0.612
DBP, mmHg, mean (SD)	63.82 (11.43)	63.21 (10.93)	63.78 (11.51)	64.17 (11.50)	64.14 (11.76)	0.321
SpO_2_, %, mean (SD)	97.27 (2.02)	97.35 (1.65)	97.28 (1.83)	97.28 (2.26)	97.18 (2.26)	0.435
Comorbidities						
CHD, n (%)	1618 (53.5)	403 (53.3)	384 (50.9)	434 (57.3)	397 (52.4)	0.080
HF, n (%)	879 (29.0)	216 (28.6)	209 (27.7)	218 (28.8)	236 (31.2)	0.483
Hypertension, n (%)	1611 (53.2)	386 (51.1)	424 (56.2)	408 (53.8)	393 (51.9)	0.198
AF, n (%)	724 (23.9)	207 (27.4)	186 (24.6)	182 (24.0)	149 (19.7)	0.005
Dyslipidemia, n (%)	689 (22.8)	153 (20.2)	176 (23.3)	175 (23.1)	185 (24.4)	0.248
DM, n (%)	828 (27.4)	68 (9.0)	135 (17.9)	235 (31.0)	390 (51.5)	< 0.001
COPD, n (%)	45 (1.5)	8 (1.1)	5 (0.7)	16 (2.1)	16 (2.1)	0.037
RF, n (%)	481 (15.9)	73 (9.7)	96 (12.7)	135 (17.8)	177 (23.4)	< 0.001
LD, n (%)	143 (4.7)	21 (2.8)	36 (4.8)	39 (5.1)	47 (6.2)	0.016
AKI^a^, n (%)	1547 (51.1)	345 (45.6)	345 (45.7)	412 (54.4)	445 (58.8)	< 0.001
CKD, n (%)	310 (10.2)	71 (9.4)	58 (7.7)	75 (9.9)	106 (14.0)	0.001
Sepsis, n (%)	162 (5.4)	24 (3.2)	32 (4.2)	40 (5.3)	66 (8.7)	< 0.001
Cancer, n (%)	360 (11.9)	98 (13.0)	103 (13.6)	88 (11.6)	71 (9.4)	0.054
Laboratory tests						
WBC, K/µL, mean (SD)	11.75 (5.88)	10.65 (4.75)	11.50 (6.86)	12.01 (5.29)	12.85 (6.18)	< 0.001
RBC, m/µL, mean (SD)	4.19 (0.73)	4.17 (0.68)	4.21 (0.71)	4.17 (0.75)	4.20 (0.78)	0.568
Platelet, K/µL, mean (SD)	252.91 (110.48)	245.85 (110.86)	254.08 (109.33)	255.96 (109.13)	255.75 (112.49)	0.236
Hemoglobin, g/dL, mean (SD)	12.69 (2.15)	12.69 (2.00)	12.77 (2.15)	12.64 (2.22)	12.67 (2.24)	0.645
Serum potassium, mEq/L, mean (SD)	4.20 (0.81)	4.19 (0.82)	4.18 (0.78)	4.16 (0.72)	4.29 (0.89)	0.007
Serum sodium, mEq/L, mean (SD)	138.43 (4.58)	138.30 (4.74)	138.69 (4.29)	138.57 (4.13)	138.15 (5.10)	0.084
TC, mg/dL, mean (SD)	163.17 (54.46)	151.66 (42.60)	160.83 (46.99)	165.97 (47.13)	175.50 (74.60)	< 0.001
TG, mg/dL, mean (SD)	143.99 (168.28)	66.55 (21.27)	104.21 (74.80)	138.64 (48.33)	266.35 (286.95)	< 0.001
LDL, mg/dL, mean (SD)	91.57 (40.10)	86.06 (35.61)	94.04 (40.53)	95.14 (40.98)	91.17 (42.83)	< 0.001
HDL, mg/dL, mean (SD)	45.78 (16.71)	52.62 (18.01)	46.43 (14.84)	43.82 (14.89)	39.42 (16.10)	< 0.001
HbA1c, %, mean (SD)	6.43 (1.59)	5.86 (0.82)	5.97 (0.79)	6.31 (1.27)	7.63 (2.32)	< 0.001
Glucose, mg/dL, mean (SD)	162.37 (105.57)	128.03 (45.85)	138.73 (47.71)	159.99 (77.66)	222.79 (169.83)	< 0.001
Albumin, g/dL, mean (SD)	3.43 (0.64)	3.49 (0.59)	3.47 (0.65)	3.40 (0.63)	3.38 (0.67)	0.013
Ucr, mg/dL, mean (SD)	97.90 (71.45)	93.62 (67.53)	100.90 (66.69)	100.79 (76.90)	96.48 (72.77)	0.745
Scr, mg/dL, mean (SD)	1.30 (1.24)	1.19 (1.06)	1.19 (1.09)	1.29 (1.31)	1.51 (1.45)	< 0.001
BUN, mg/dL, mean (SD)	24.31 (17.61)	21.94 (12.78)	22.66 (17.55)	24.74 (16.94)	27.91 (21.49)	< 0.001
Uric acid, mg/dL, mean (SD)	5.66 (2.86)	5.74 (2.89)	4.93 (1.90)	5.58 (3.05)	6.01 (3.10)	0.377
TyG index, mean (SD)	9.16 (0.74)	8.30 (0.33)	8.87 (0.12)	9.32 (0.15)	10.13 (0.49)	< 0.001
Events						
LOS ICU, days, mean (SD)	4.28 (6.08)	3.68 (6.38)	3.78 (5.35)	4.16 (4.95)	5.49 (7.21)	< 0.001
LOS Hospital, days, mean (SD)	8.71 (10.12)	7.68 (8.71)	7.83 (8.58)	8.82 (9.71)	10.50 (12.68)	< 0.001
ICU death, n (%)	258 (8.5)	42 (5.6)	59 (7.8)	77 (10.2)	80 (10.6)	0.001
Hospital death, n (%)	350 (11.6)	57 (7.5)	85 (11.3)	102 (13.5)	106 (14.0)	< 0.001
Follow-up death, n (%)	1148 (37.9)	265 (35.1)	270 (35.8)	306 (40.4)	307 (40.6)	0.040

TyG index: Q1 (6.23–8.65), Q2 (8.65–9.08), Q3 (9.08–9.59), Q4 (9.59–12.43)

*TyG index* triglyceride glucose index, *BMI* body mass index, *ICU* intensive care unit, *SOFA* sequential organ failure assessment, *LODS* logistic organ dysfunction system, *OASIS* Oxford acute severity of illness, *SIRS* systemic inflammatory response syndrome, *APSIII* acute physiology score III, *SAPSII* simplified acute physiological score II, *CCU* coronary care unit, *CSRU* cardiac surgery recovery unit, *MICU* medical intensive care unit, *SICU* surgical intensive care unit, *TSICU* trauma/surgical intensive care unit, *HR* heart rate, *bmp* beats per minute, *SBP* systolic blood pressure, *DBP* diastolic blood pressure, *SpO*_*2*_ pulse blood oxygen saturation, *CHD* coronary heart disease, *HF* heart failure, *AF* atrial fibrillation, *DM* diabetes mellitus, *COPD* chronic obstructive pulmonary disease, *RF* respiratory failure, *LD* liver disease, *AKI* acute kidney injury, *CKD* chronic kidney disease, *WBC* white blood cell, *RBC* red blood cell, *TC* total cholesterol, *TG* triglyceride, *LDL* low-density lipoprotein, *HDL* high-density lipoprotein, *HbA1c* hemoglobin A1c, *Ucr* urine creatinine, *Scr* serum creatinine, *BUN* blood urea nitrogen, *LOS* length of stay

^a^AKI was defined according to KDIGO guidelines as an increase in serum creatinine (Scr) by ≥ 0.3 mg/dL (≥ 26.5 μmol/L) from baseline within 48 h

### Incidence rate of all-cause mortality among different groups

**Fig. 2:**
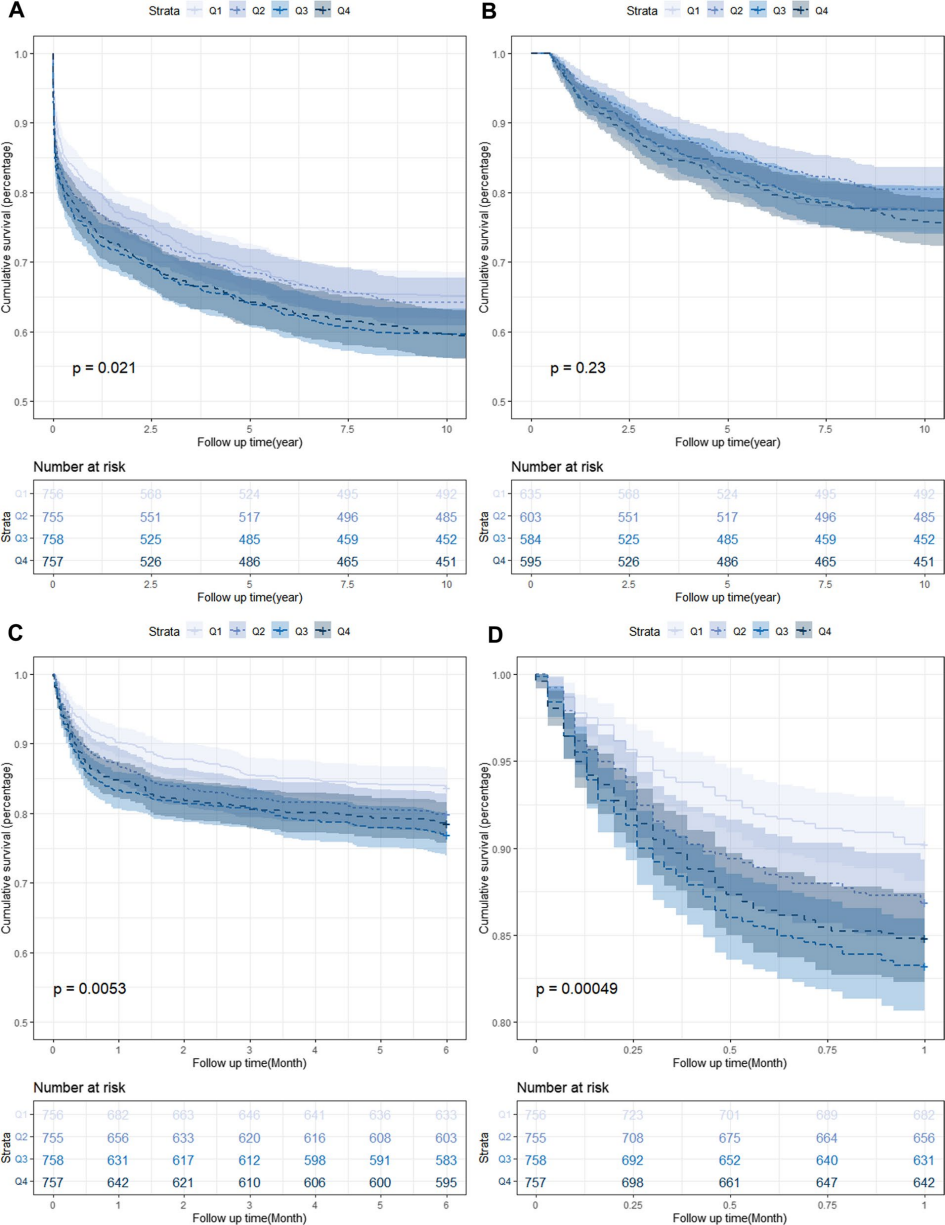
Kaplan–Meier survival analysis curves for all-cause mortality. TyG index: Q1 (6.23–8.65), Q2 (8.65–9.08), Q3 (9.08–9.59), Q4 (9.59–12.43). Kaplan–Meier curves showing cumulative probability of all-cause mortality according to groups at 10 years (**A**), landmark analysis from 6 months to 10 years (**B**), landmark analysis at 6 months (**C**), and Kaplan–Meier survival analysis curves for all-cause mortality according to groups at 1 month (**D**)

The Kaplan–Meier survival analysis curves for assessing the incidence of all-cause mortality among groups based on the quartile groupings of the TyG index are shown in Fig. 2. There was a statistically significant difference in mortality rate in the groups (Q1: 35.1% vs. Q2: 35.8% vs. Q3: 40.4% vs. Q4: 40.6%, log-rank *P* = 0.021, Fig. 2A). Higher mortality driven by high TyG index disappeared after 6 months, and no difference was found among groups (log-rank *P* = 0.230, Fig. 2B). Applying the landmark method, during the 6 months of follow-up, the all-cause mortality of patients with higher TyG index was obvious higher than lower TyG index (Q1: 16.3% vs. Q2: 19.8% vs. Q3: 23.1% vs. Q4: 21.4%, log-rank *P* = 0.005, Fig. 2C). Notably, more significantly result was observed during the short-term follow-up of 1 month (Q1: 9.8% vs. Q2: 13.1% vs. Q3: 16.8% vs. Q4: 15.2%, Log-rank *P* < 0.001, Fig. 2D).

### Association between the all-cause mortality and TyG index

**Fig. 3:**
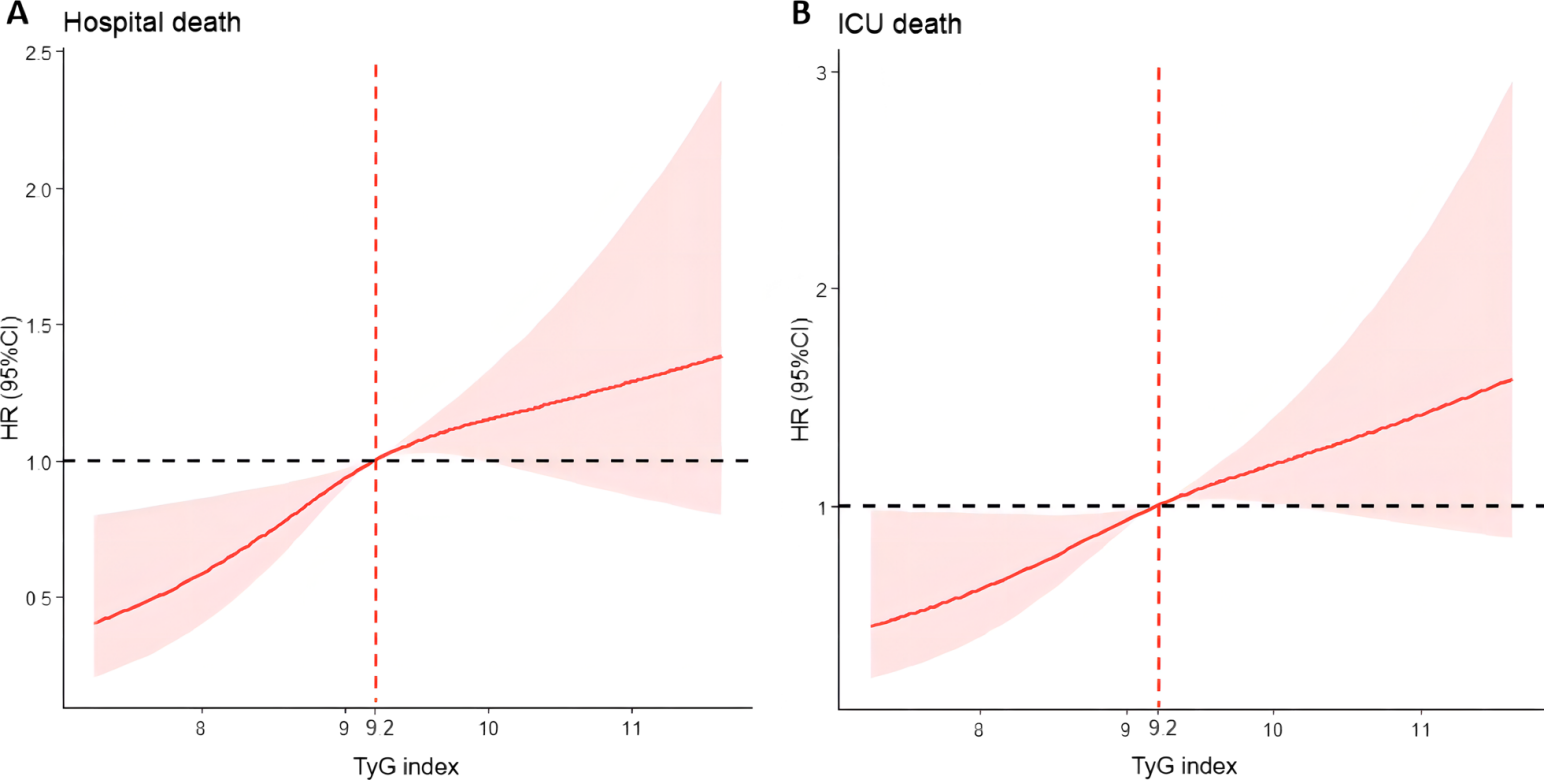
Restricted cubic spline regression analysis of TyG index with in hospital all-cause mortality. Heavy central lines represent the estimated adjusted hazard ratios, with shaded ribbons denoting 95% confidence intervals. TyG index 9.2 was selected as the reference level represented by the vertical dotted lines. The horizontal dotted lines represent the hazard ratio of 1.0. **A** Restricted cubic spline for hospital death. **B** Restricted cubic spline for ICU death. *HR* hazard ratio, *CI* confidence interval, *TyG* triglyceride-glucose

Cox proportional risk analysis showed the significant association between TyG index and hospital death both in unadjusted model (HR, 1.29 [95% CI 1.12–1.48] *P* < 0.001) and fully adjusted model (HR, 2.19 [95% CI 1.59–3.03] *P* < 0.001). Furthermore, TyG index was also associated ICU death in unadjusted model (HR, 1.31 [95% CI 1.12–1.53] *P* < 0.001) and fully adjusted model (HR, 1.72 [95% CI 1.18–2.52] *P* = 0.005). The risk of hospital death of TyG index Q2, Q3 and Q4 was higher than TyG index Q1, and showed a tendency of increasing with the TyG index (Q1 vs. Q2: HR, 1.70 [95% CI 1.06–2.70]; Q3: HR, 2.08 [95% CI 1.29–3.33]; Q4: HR, 2.80 [95% CI 1.53–5.13]; *P* for trend < 0.001). Similar results were obtained in Cox proportional risk analysis of the TyG index and ICU death (Q1 vs. Q2: HR, 1.52 [95% CI 0.88–2.64]; Q3: HR, 1.88 [95% CI 1.09–3.24]; Q4: HR, 1.95 [95% CI 0.96–3.99]; *P* for trend = 0.036) (Table 2). The RCS regression model revealed that higher levels of TyG index (> 9.2) was associated with an increased risk of hospital death and ICU death (Fig. 3).

**Table 2:** Cox proportional hazard ratios (HR) for all-cause mortality

Categories	Events (%)	Model 1			Model 2			Model 3		
		HR (95% CI)	*P*-value	*P* for trend	HR (95% CI)	*P*-value	*P* for trend	HR (95% CI)	*P*-value	*P* for trend
Hospital death										
Continuous variable per 1 unit		1.29 (1.12–1.48)	< 0.001		1.26 (1.09–1.46)	0.002		2.19 (1.59–3.03)	< 0.001	
Quartile^a^	350 (11.57)			< 0.001			0.004			< 0.001
Q1 (N = 756)	57 (7.54)	Ref.			Ref.			Ref.		
Q2 (N = 755)	85 (11.26)	1.57 (1.12–2.20)	0.008		1.57 (1.12–2.21)	0.009		1.70 (1.06–2.70)	0.027	
Q3 (N = 758)	102 (13.46)	1.73 (1.25–2.39)	< 0.001		1.69 (1.22–2.35)	0.002		2.08 (1.29–3.33)	0.002	
Q4 (N = 757)	106 (14.00)	1.75 (1.27–2.42)	< 0.001		1.69 (1.20–2.37)	0.003		2.80 (1.53–5.13)	< 0.001	
ICU death										
Continuous variable per 1 unit		1.31 (1.12–1.53)	< 0.001		1.21 (1.02–1.43)	0.029		1.72 (1.18–2.52)	0.005	
Quartile	258 (8.53)			0.002			0.030			0.036
Q1 (N = 756)	42 (5.56)	Ref.			Ref.			Ref.		
Q2 (N = 755)	59 (7.81)	1.46 (0.98–2.17)	0.060		1.44 (0.96–2.15)	0.074		1.52 (0.88–2.64)	0.136	
Q3 (N = 758)	77 (10.16)	1.75 (1.20–2.54)	0.004		1.65 (1.13–2.42)	0.010		1.88 (1.09–3.24)	0.023	
Q4 (N = 757)	80 (10.57)	1.78 (1.22–2.58)	0.003		1.55 (1.04–2.29)	0.030		1.95 (0.96–3.99)	0.066	

Model 1: unadjusted

Model 2: adjusted for age, sex, ethnicity, first care unit

Model 3: adjusted for age, sex, ethnicity, first care unit, SOFA score, LODS score, white blood cell, red blood cell, hemoglobin, serum sodium, serum potassium, total cholesterol, low-density lipoprotein, high-density lipoprotein, albumin, serum creatinine, coronary heart disease, heart failure, hypertension, dyslipidemia, diabetes, chronic obstructive pulmonary disease, respiratory failure, liver disease, chronic kidney disease, acute kidney injury, sepsis, cancer

^a^TyG index: Q1 (6.23–8.65), Q2 (8.65–9.08), Q3 (9.08–9.59), Q4 (9.59–12.43)

**Fig. 4:**
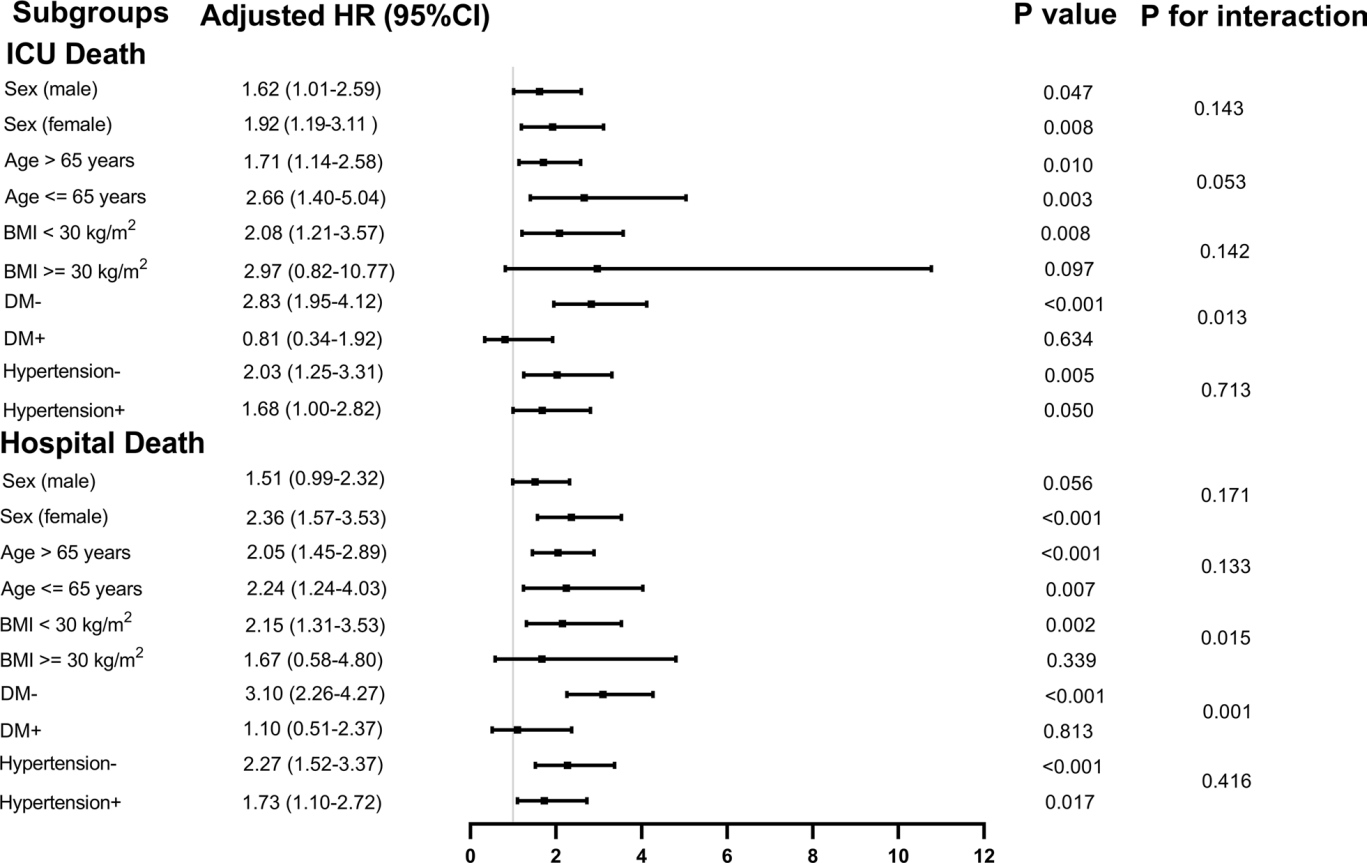
Forest plots of hazard ratios for the primary outcome in different subgroups. *HR* hazard ratio, *CI* confidence interval, *TyG* triglyceride-glucose, *ICU* intensive care unit, *BMI* body mass index, *DM* diabetes mellitus

In addition, we conducted a stratified analyses of the relationship between the TyG index and all-cause mortality according to the potential modifiers, including sex, age, BMI, DM and hypertension (Fig. 4). And the TyG index was significantly associated with an increased risk of ICU death in a subgroup of female [HR (95% CI) 1.92 (1.19–3.11)], male [HR (95% CI) 1.62 (1.01–2.59)], those aged ≤ 65 years [HR (95% CI) 2.66 (1.40–5.04)] or > 65 years [HR (95% CI) 1.71 (1.14–2.58)], those with BMI < 30 kg/m^2^ [HR (95% CI) 2.08 (1.21–3.57)], those without DM [HR (95% CI) 2.83 (1.95–4.12)], and those without hypertension [HR (95% CI) 2.03 (1.25–3.31)] (all P < 0.05). Interestingly, the predictive value of TyG index seemed to be more prominent in patients without DM [HR (95% CI) without DM 2.83 (1.95–4.12) vs. with DM 0.81 (0.34–1.92), *P* for interaction = 0.013]. Similar results were obtained in stratified analyses of the TyG index and hospital death (Fig. 4).

### Incremental effect of TyG index on predictive value for primary outcomes

The TyG index had an incremental effect on the AUC of existing severity of illness scores to predict all-cause mortality, including SOFA score (hospital: 0.765 vs. 0.772; ICU: 0.811 vs. 0.815), LODS score (hospital: 0.791 vs. 0.796; ICU: 0.834 vs. 0.837), OASIS score (hospital: 0.798 vs. 0.801; ICU: 0.810 vs. 0.812), SIRS score (hospital: 0.689 vs. 0.706; ICU: 0.708 vs. 0.722), APSIII (hospital: 0.794 vs. 0.798; ICU: 0.829 vs. 0.831) and SAPSII (hospital: 0.834 vs. 0.838; ICU: 0.854 vs. 0.856). All *P*-values < 0.001 (Table 3).

**Table 3:** Discrimination of each predictive model for outcomes using AUC

Models	AUC (95% CI)	*P*-value	Models	AUC (95% CI)	*P*-value
Hospital death					
SOFA Score	0.765 (0.734–0.797)	< 0.001	+ TyG index	0.772 (0.742–0.802)	< 0.001
LODS Score	0.791 (0.763–0.820)	< 0.001	+ TyG index	0.796 (0.768–0.824)	< 0.001
OASIS Score	0.798 (0.771–0.824)	< 0.001	+ TyG index	0.801 (0.774–0.828)	< 0.001
SIRS Score	0.689 (0.659–0.720)	< 0.001	+ TyG index	0.706 (0.676–0.736)	< 0.001
APSIII	0.794 (0.766–0.822)	< 0.001	+ TyG index	0.798 (0.771–0.825)	< 0.001
SAPSII	0.834 (0.811–0.858)	< 0.001	+ TyG index	0.838 (0.815–0.861)	< 0.001
ICU death					
SOFA Score	0.811 (0.780–0.842)	< 0.001	+ TyG index	0.815 (0.785–0.846)	< 0.001
LODS Score	0.834 (0.806–0.862)	< 0.001	+ TyG index	0.837 (0.810–0.865)	< 0.001
OASIS Score	0.810 (0.780–0.840)	< 0.001	+ TyG index	0.812 (0.782–0.842)	< 0.001
SIRS Score	0.708 (0.675–0.742)	< 0.001	+ TyG index	0.722 (0.690–0.755)	< 0.001
APSIII	0.829 (0.800–0.857)	< 0.001	+ TyG index	0.831 (0.803–0.858)	< 0.001
SAPSII	0.854 (0.828–0.879)	< 0.001	+ TyG index	0.856 (0.831–0.881)	< 0.001

*AUC* area under curve, *SOFA* sequential organ failure assessment, *LODS* logistic organ dysfunction system, *OASIS* Oxford acute severity of illness score, *SIRS* systemic inflammatory response syndrome, *APSIII* acute physiology score III, *SAPSII*, simplified acute physiological score II, *TyG index* triglyceride-glucose index, *ICU* intensive care unit

### Relationship between TyG index and length of stay

Multiple linear regression analysis showed that the TyG index was positively associated with length of stay even in the fully adjusted model among patients who survived the hospital stay (*β* = 1.36, *P* = 0.008) and those who survived the ICU stay (*β* = 0.87, *P* = 0.004, Table 4).

**Table 4:** Relationship between TyG index and length of stay (LOS)

	Coef.	S. E	t-value	*P*-value
LOS hospital^a^				
Model 1	1.66	0.25	6.65	< 0.001
Model 2	1.55	0.25	6.25	< 0.001
Model 3	1.36	0.51	2.68	0.008
LOS ICU^b^				
Model 1	0.96	0.14	6.91	< 0.001
Model 2	0.91	0.14	6.46	< 0.001
Model 3	0.87	0.30	2.87	0.004

Model 1: unadjusted

Model 2: adjusted for age, sex ethnicity, first care unit

Model 3: adjusted for age, sex, ethnicity, first care unit, SOFA score, LODS score, white blood cell, red blood cell, hemoglobin, serum sodium, serum potassium, total cholesterol, low-density lipoprotein, high-density lipoprotein, albumin, serum creatinine, coronary heart disease, heart failure, hypertension, dyslipidemia, diabetes, chronic obstructive pulmonary disease, respiratory failure, liver disease, chronic kidney disease, acute kidney injury, sepsis, cancer

^a^The relationship between TyG index and length of hospital stay was analyzed in a cohort that survived the hospital stay (n = 2676)

^b^The relationship between TyG index and length of ICU stay was analyzed in a cohort that survived the ICU stay (n = 2768)

## Discussion

To the best of our knowledge, this study was the first to explore the relationship between TyG index and all-cause mortality in ICU patients. In the present study, the information of unselected ICU adult patients were extracted from the MIMIC III database, the primary finding is that increased TyG index was a strong independent predictor of greater mortality in ICU patients. This association remained after adjustment for a wide variety of clinical and laboratory variables. Most importantly, this study provides a novel, simple and efficient biomarker for the early diagnosis of IR in critically ill patients.

The severity of IR in vivo can be determined using the hyperinsulinaemic–euglycaemic clamp technique which is the gold standard for evaluating IR [22]. However, the performance of the hyperinsulinaemic–euglycaemic clamp technique is time-consuming, costly and complex [14]. Alternatives for estimating IR include the Quantitative Insulin Sensitivity Check Index (QUICKI) and the Homeostasis model assessment of IR (HOMA-IR) [23, 24]. However, constrained to the complex mathematical calculation or the requirement of insulin concentration examination, the clinical popularity of QUICKI and HOMA-IR remain challenging. Recently, the TyG index, based on the FBG and triglycerides, is a novel index that has been well-recognized as a simple and reliable surrogate of IR [14]. Former studies have proved that TyG index has high correlation with hyperinsulinaemic–euglycaemic clamp, either in individuals with or without DM [16, 25]. In addition, compared with the HOMA-IR, TyG index showed better evaluation efficiency [26, 27]. Among them, serum triglyceride and glucose are low-cost routine biochemical detection items [6], therefore, the immense advantage of using such a simple method of IR identification is obviously that it is easily accessible in any clinical settings and has a good application prospect [28].

Recent studies have widely used the TyG index as a marker of IR [29]. Previous studies conducted in Asia and Europe validated the strong association between TyG index and incidence of DM, suggesting that TyG index might be an important predictor of early identification of individuals at high risk for diabetes and prediabetes, even better than other risk factors such as fasting glucose and triglycerides [30–32]. Zhao et al. [33] and Chiu et al. [34] showed that TyG index was associated with macrovascular and microvascular damage in both elderly community-dwelling Chinese population and diabetic population. Hu et al. [35] found that, regardless of diabetes status, patients with high TyG index had a significantly higher risk of cardiovascular events. For patients with ACS undergoing percutaneous coronary intervention, TyG index might be a better predictor of cardiovascular risk than FBG or glycated hemoglobin. A recent study demonstrated that the TyG index was directly correlated with poor prognosis in patients with acute decompensated heart failure regardless of DM status [17]. Furthermore, Liu et al. [36] found that elevated level of TyG index reflected a more severe IR and was non-linear associated with all-cause and cardiovascular mortality in the general population. However, current data about associations between TyG index and critically ill patients are limited. Zhang et al. [20] demonstrated that TyG index was a potential predictor of hospital and ICU mortality in a study involving only patients with critical stroke. Additionally, in our study of unselected ICU adult patients, we found that TyG index was an independent predictor of hospitalization and ICU mortality in critically ill patients, which makes the study be great agreement and complement to previous literature [12], which considered that IR was related to the severity of their condition rather than the different admission diagnoses of ICU patients.

IR is defined as unresponsiveness of anabolic processes to the normal effects of insulin, and it has been postulated that many metabolic abnormalities associated with critical illness are related to a loss of tissue sensitivity to insulin [8, 12], and which is not already reflected in severity scores [37]. Severity of illness scores and their use in predicting outcomes have gained considerable favor worldwide and have been proven effective for predicting mortality in ICU patients [4, 38, 39]. However, whether the addition of TyG index has an incremental effect on the prediction of all-cause mortality in ICU patients at the basis of severity scores is uncertain. This study revealed a significant prognostic impact of the TyG index and its incremental effect on risk stratification based on severity scores in critically ill patients. In addition, some of these scores encompass various clinical information including patients’ symptoms, signs, laboratory tests, microbiology findings. In the absence of any of this information, these scores cannot be used [4]. Compared with above scores, the TyG index is an easily available, inexpensive and reliable test [6], and could be used as an independent predictor of hospitalization and ICU mortality in critically ill patients. Therefore, routine assessment of TyG index may improve risk stratification and facilitate decision making in ICU patients.

Intriguingly, some previous studies have found that insulin treatment and lipid-lowering drugs were not associated with TyG index in non-critically ill patients. They believe that the above unexpected results may be related to the history of insulin treatment, and lipid-lowering drugs can not directly reflect the observed level of TyG index [40, 41]. However, our current study found that the predictive value of IR evaluated by TyG index seemed to be more prominent in patients without DM [HR (95% CI) without DM 2.83 (1.95–4.12) vs. with DM 0.81 (0.34–1.92), *P* for interaction = 0.013], suggesting that antidiabetic treatment may have an important effect on the predictive performance of TyG index for adverse clinical outcomes. This inconsistency may be due to the fact that critically ill diabetic patients are more likely to receive intensive insulin therapy, Van den Berghe et al. [42] showed that intensive insulin therapy reduced mortality during intensive care from 8.0 percent with conventional treatment to 4.6 percent. Another important finding of our study was that patients with higher TyG index were younger, and the relationship between TyG index and all-cause mortality seemed to be more pronounced in younger patients, which was consistent with the previous study [14]. Contrary to conventional wisdom, clinicians may pay more attention to older patients because they may have more comorbidities, whereas our study calls for the same attention to be given to younger patients because they may have a higher mortality rate.

IR is a clinical condition characterized by impaired glucose processing in the presence of normal or elevated serum insulin concentrations [7, 8]. The development of IR was no disease-specific reaction but a prevailing response to critical illness [12]. Although the mechanism underlying the close connection between the TyG index and all-cause mortality in ICU patients has not been elucidated, it might be attributed to the relationship between the IR status represented by the TyG index and the severity of the disease. First, IR has been widely demonstrated to be well correlated with endothelial dysfunction, oxidative stress, cardiovascular remodeling, coagulation imbalance and inflammation response [43–45], and all of which were important reasons for the aggravation of ICU patients. Second, the relationship between severity of IR and illness could be explained by an increased productions of serum cytokines. These productions have been shown to increase with disease severity, and as the key components of systemic inflammation and stress response induced IR [8, 46]. Third, hyperglycemia associated with IR was common in critically ill patients, even in those without diabetes previously [42]. Hyperglycemia promoted tissue acidosis, production of reactive oxygen species and nitrogen, and inflammatory cell infiltration, leading to more severe tissue structural dysfunction. Fourth, IR was associated with macrovascular disease, neuropathy, and organ failure [13], which led the continued deterioration in critically ill patients ultimately. Studies have shown that FBG levels mainly reflected IR from the liver, whereas fasting triglycerides levels mainly reflected IR from adipose cells. Therefore, the TyG index might reflect IR from two aspects and thus be closely related to IR [47, 48]. Furthermore, a recent review showed that it would be interesting to explore whether a postprandial TyG index might have clinical significance, because increased postprandial levels of triglyceride and glucose are metabolically abnormal responses to IR [49].

Our study confirmed that TyG index could be used as an effective predictor in clinical practice, and was independent risk predictor of ICU death and hospital death. However, we must acknowledge some limitations. Firstly, this was a retrospective analysis derived from an observational study, which could not definitively establish causality, but we have carried out careful, multifaceted and rigorous statistical methods to produce valid and reliable and results. Further studies need to be performed to determine whether interventions for TyG index have a positive impact on improving clinical prognosis. Secondly, due to the limitation of the database, there is no way to confirm that all glucose and lipids are the results of fasting. Thirdly, the data were from the United States, and thus the results might not be completely applicable to ICUs in other countries, but the enrolled patients came from different races, therefore, it had a certain representative.

## Conclusions

Increased IR extent presented by TyG index is a prominent risk predictor of all-cause mortality in ICU patients. Our findings indicate that this simple index facilitates early identification of IR in critically ill patients, which can improve risk stratification and guide subsequent interventions. All of these findings strongly support the importance of including TyG index in physicians’ daily work. Further prospective studies are required to confirm our findings.

## Supplementary Information

**Additional file 1: Table S1.** Comparisons of baseline characteristics between the original cohort and matched cohort.

## Data Availability

The datasets generated and analyzed during the current study are available from the corresponding author on reasonable request.

## Abbreviations

ICU: Intensive care unit
TyG: Triglyceride-glucose
IR: Insulin resistance
FBG: Fasting blood glucose
ACS: Acute coronary syndrome
HF: Heart failure
MIMIC-III: Medical Information Mart for Intensive Care III
MIT: Massachusetts Institute of Technology
BIDMC: Beth Israel Deaconess Medical Center
SOFA: Sequential organ failure assessment
LODS: Logistic organ dysfunction system
SIRS: Systemic inflammatory response syndrome
OASIS: Overall anxiety severity and impairment scale
APSIII: Acute physiology score III
SAPSII: Simplified acute physiological score II
CHD: Coronary heart disease
AF: Atrial fibrillation
DM: Diabetes mellitus
COPD: Chronic obstructive pulmonary disease
RF: Respiratory failure
LD: Liver disease
CKD: Chronic kidney disease
AKI: Acute kidney injury
KDIGO: Kidney Disease: Improving Global Outcomes
Scr: Serum creatinine
SD: Standard deviation
IQR: Interquartile range
PSM: Propensity score matching
HR: Hazard ratio
CI: Confidence interval
RCS: Restricted cubic spline
BMI: Body mass index
VIF: Variance inflation factor
WBC: White blood cell
RBC: Red blood cell
TC: Total cholesterol
LDL: Low-density lipoprotein
HDL: High-density lipoprotein
AUC: Area under the curve
QUICKI: Quantitative Insulin Sensitivity Check Index
HOMA-IR: Homeostasis model assessment of IR
