# Supplementary material for: Triglyceride-glucose index linked to all-cause mortality in critically ill patients: a cohort of 3026 patients
Source: Cardiovasc Diabetol. 2022 Jul 8;21:128. doi: 10.1186/s12933-022-01563-z (PMC9270811; doi:10.1186/s12933-022-01563-z)
Supplement: Supplementary file 1 — Additional file 1: Table S1. Comparisons of baseline characteristics between the original cohort and matched cohort. [file 12933_2022_1563_MOESM1_ESM.docx]

**Table S1. Comparisons of Baseline Characteristics between the Original Cohort and Matched Cohort ^a^**

| **Characteristic** | **Original Cohort** | | | | **Matched Cohort** | | | |
| --- | --- | --- | --- | --- | --- | --- | --- | --- |
|  | **Overall** | **Lower TyG** | **Higher TyG** | ***P-value*** | **Overall** | **Lower TyG** | **Higher TyG** | ***P-value*** |
|  | (N=3,026) | (N=1,636) | (N=1,390) |  | (N=2,600) | (N=1,300) | (N=1,300) |  |
|  | **Demographic** | | | | | | | |
| Age, years, mean (SD) | 65.44 (16.07) | 67.56 (16.08) | 62.94 (15.71) | <0.001 | 64.34 (15.38) | 64.43 (15.69) | 64.25 (15.08) | 0.769 |
| Male, n (%) | 1786 (59.0) | 964 (58.9) | 822 (59.1) | 0.935 | 1524 (58.6) | 763 (58.7) | 761 (58.5) | 0.968 |
| Ethnicity, n (%) |  |  |  | 0.136 |  |  |  | 0.781 |
| Asian | 58 (1.9) | 33 (2.0) | 25 (1.8) |  | 44 (1.7) | 19 (1.5) | 25 (1.9) |  |
| Black | 202 (6.7) | 110 (6.7) | 92 (6.6) |  | 174 (6.7) | 84 (6.5) | 90 (6.9) |  |
| White | 2066 (68.3) | 1144 (69.9) | 922 (66.3) |  | 1758 (67.6) | 892 (68.6) | 866 (66.6) |  |
| Hispanic/Latino | 83 (2.7) | 44 (2.7) | 39 (2.8) |  | 71 (2.7) | 34 (2.6) | 37 (2.8) |  |
| Other | 617 (20.4) | 305 (18.6) | 312 (22.4) |  | 553 (21.3) | 271 (20.8) | 282 (21.7) |  |
| Weight, kg, mean (SD) | 82.25 (22.71) | 78.77 (21.20) | 86.25 (23.71) | <0.001 | 82.95 (22.46) | 80.27 (21.89) | 85.60 (22.72) | <0.001 |
| Height, cm, mean (SD) | 169.92 (14.47) | 170.17 (16.68) | 169.66 (11.72) | 0.503 | 170.00 (14.60) | 170.54 (17.09) | 169.50 (11.80) | 0.202 |
| BMI, kg/m^2^, mean (SD) | 31.35 (76.35) | 32.29 (105.99) | 30.36 (12.90) | 0.630 | 31.76 (80.83) | 33.42 (115.70) | 30.22 (12.90) | 0.476 |
|  | **ICU Admission** | | | | | | | |
| SOFA score, mean (SD) | 3.32 (2.91) | 2.97 (2.54) | 3.74 (3.25) | <0.001 | 3.30 (2.91) | 2.95 (2.64) | 3.66 (3.11) | <0.001 |
| LODS score, mean (SD) | 3.49 (2.56) | 3.18 (2.27) | 3.85 (2.82) | <0.001 | 3.48 (2.58) | 3.13 (2.30) | 3.84 (2.79) | <0.001 |
| OASIS score, mean (SD) | 30.94 (8.96) | 30.28 (8.44) | 31.72 (9.47) | <0.001 | 30.66 (9.01) | 29.57 (8.41) | 31.75 (9.45) | <0.001 |
| SIRS score, mean (SD) | 2.51 (1.06) | 2.38 (1.05) | 2.65 (1.05) | <0.001 | 2.52 (1.07) | 2.41 (1.07) | 2.63 (1.06) | <0.001 |
| APSIII, mean (SD) | 39.29 (18.74) | 37.04 (16.76) | 41.93 (20.52) | <0.001 | 39.10 (18.86) | 36.44 (16.85) | 41.76 (20.34) | <0.001 |
| SAPSII, mean (SD) | 32.83 (13.23) | 31.97 (12.19) | 33.85 (14.29) | <0.001 | 32.47 (13.37) | 30.82 (12.27) | 34.12 (14.20) | <0.001 |
| First Care Unit, n (%) |  |  |  | <0.001 |  |  |  | 0.957 |
| CCU | 1259 (41.6) | 689 (42.1) | 570 (41.0) |  | 1137 (43.7) | 577 (44.4) | 560 (43.1) |  |
| CSRU | 152 (5.0) | 69 (4.2) | 83 (6.0) |  | 139 (5.3) | 68 (5.2) | 71 (5.5) |  |
| MICU | 708 (23.4) | 345 (21.1) | 363 (26.1) |  | 602 (23.2) | 299 (23.0) | 303 (23.3) |  |
| SICU | 643 (21.2) | 381 (23.3) | 262 (18.8) |  | 510 (19.6) | 254 (19.5) | 256 (19.7) |  |
| TSICU | 264 (8.7) | 152 (9.3) | 112 (8.1) |  | 212 (8.2) | 102 (7.8) | 110 (8.5) |  |
|  | **Vital Signs** | | | | | | | |
| HR, bmp, mean (SD) | 81.13 (16.20) | 79.09 (15.46) | 83.54 (16.71) | <0.001 | 81.35 (16.00) | 79.85 (15.59) | 82.85 (16.28) | <0.001 |
| SBP, mmHg, mean (SD) | 123.12 (18.63) | 122.85 (18.71) | 123.44 (18.54) | 0.386 | 122.50 (18.52) | 121.45 (18.30) | 123.55 (18.68) | 0.004 |
| DBP, mmHg, mean (SD) | 63.82 (11.43) | 63.51 (11.19) | 64.20 (11.70) | 0.098 | 63.96 (11.47) | 64.03 (11.34) | 63.90 (11.60) | 0.766 |
| SpO_2_, %, mean (SD) | 97.27 (2.02) | 97.30 (1.75) | 97.24 (2.30) | 0.385 | 97.27 (2.06) | 97.27 (1.76) | 97.27 (2.33) | 1.000 |
|  | **Comorbidities** | | | | | | | |
| CHD, n (%) | 1618 (53.5) | 861 (52.6) | 757 (54.5) | 0.332 | 1425 (54.8) | 694 (53.4) | 731 (56.2) | 0.156 |
| HF, n (%) | 879 (29.0) | 469 (28.7) | 410 (29.5) | 0.645 | 753 (29.0) | 358 (27.5) | 395 (30.4) | 0.120 |
| Hypertension, n (%) | 1611 (53.2) | 871 (53.2) | 740 (53.2) | 1.000 | 1376 (52.9) | 667 (51.3) | 709 (54.5) | 0.107 |
| AF, n (%) | 724 (23.9) | 420 (25.7) | 304 (21.9) | 0.016 | 604 (23.2) | 306 (23.5) | 298 (22.9) | 0.745 |
| Dyslipidemia, n (%) | 689 (22.8) | 359 (21.9) | 330 (23.7) | 0.258 | 595 (22.9) | 278 (21.4) | 317 (24.4) | 0.076 |
| DM, n (%) | 828 (27.4) | 230 (14.1) | 598 (43.0) | <0.001 | 751 (28.9) | 180 (13.8) | 571 (43.9) | <0.001 |
| COPD, n (%) | 45 (1.5) | 16 (1.0) | 29 (2.1) | 0.018 | 42 (1.6) | 14 (1.1) | 28 (2.2) | 0.043 |
| RF, n (%) | 481 (15.9) | 193 (11.8) | 288 (20.7) | <0.001 | 408 (15.7) | 153 (11.8) | 255 (19.6) | <0.001 |
| LD, n (%) | 143 (4.7) | 65 (4.0) | 78 (5.6) | 0.042 | 122 (4.7) | 59 (4.5) | 63 (4.8) | 0.781 |
| AKI **^b^**, n (%) | 1547 (51.1) | 761 (46.5) | 786 (56.5) | <0.001 | 1334 (51.3) | 600 (46.2) | 734 (56.5) | <0.001 |
| CKD, n (%) | 310 (10.2) | 138 (8.4) | 172 (12.4) | <0.001 | 257 (9.9) | 97 (7.5) | 160 (12.3) | <0.001 |
| Sepsis, n (%) | 162 (5.4) | 58 (3.5) | 104 (7.5) | <0.001 | 136 (5.2) | 44 (3.4) | 92 (7.1) | <0.001 |
| Cancer, n (%) | 360 (11.9) | 222 (13.6) | 138 (9.9) | 0.002 | 292 (11.2) | 159 (12.2) | 133 (10.2) | 0.120 |
|  | **Laboratory Tests** | | | | | | | |
| WBC, K/uL, mean (SD) | 11.75 (5.88) | 11.14 (5.86) | 12.48 (5.82) | <0.001 | 11.88 (6.00) | 11.35 (6.19) | 12.41 (5.74) | <0.001 |
| RBC, m/uL, mean (SD) | 4.19 (0.73) | 4.19 (0.69) | 4.18 (0.77) | 0.640 | 4.20 (0.73) | 4.21 (0.70) | 4.19 (0.76) | 0.506 |
| Platelet, K/uL, mean (SD) | 252.91 (110.48) | 250.89 (110.08) | 255.29 (110.94) | 0.276 | 254.16 (110.30) | 252.55 (111.90) | 255.77 (108.69) | 0.456 |
| Hemoglobin, g/dL, mean (SD) | 12.69 (2.15) | 12.74 (2.08) | 12.63 (2.24) | 0.156 | 12.72 (2.16) | 12.78 (2.11) | 12.66 (2.21) | 0.138 |
| Potassium, mEq/L, mean (SD) | 4.20 (0.81) | 4.18 (0.79) | 4.23 (0.82) | 0.073 | 4.20 (0.81) | 4.15 (0.78) | 4.24 (0.82) | 0.005 |
| Sodium, mEq/L, mean (SD) | 138.43 (4.58) | 138.49 (4.46) | 138.35 (4.72) | 0.432 | 138.42 (4.60) | 138.47 (4.50) | 138.36 (4.69) | 0.533 |
| TC, mg/dL, mean (SD) | 163.17 (54.46) | 156.93 (45.02) | 170.95 (63.49) | <0.001 | 164.59 (55.98) | 158.47 (46.67) | 170.92 (63.61) | <0.001 |
| TG, mg/dL, mean (SD) | 143.99 (168.28) | 87.82 (57.40) | 210.09 (222.94) | <0.001 | 148.99 (177.17) | 89.57 (62.69) | 208.41 (227.60) | <0.001 |
| LDL, mg/dL, mean (SD) | 91.57 (40.10) | 90.48 (38.26) | 92.96 (42.31) | 0.121 | 92.50 (40.56) | 92.30 (39.68) | 92.70 (41.49) | 0.818 |
| HDL, mg/dL, mean (SD) | 45.78 (16.71) | 49.43 (16.64) | 41.18 (15.64) | <0.001 | 45.26 (16.55) | 48.93 (16.70) | 41.43 (15.50) | <0.001 |
| HbA1c, %, mean (SD) | 6.43 (1.59) | 5.92 (0.82) | 7.05 (2.03) | <0.001 | 6.50 (1.64) | 5.94 (0.87) | 7.03 (1.99) | <0.001 |
| Glucose, mg/dL, mean (SD) | 162.37 (105.57) | 134.76 (47.91) | 194.92 (140.09) | <0.001 | 165.04 (109.39) | 134.45 (47.64) | 195.68 (140.74) | <0.001 |
| Albumin, g/dL, mean (SD) | 3.43 (0.64) | 3.48 (0.62) | 3.38 (0.66) | 0.002 | 3.43 (0.65) | 3.47 (0.63) | 3.39 (0.66) | 0.019 |
| Ucr, mg/dL, mean (SD) | 97.90 (71.45) | 95.90 (65.86) | 99.60 (75.90) | 0.485 | 98.45 (73.05) | 95.41 (66.89) | 100.69 (77.27) | 0.368 |
| Scr, mg/dL, mean (SD) | 1.30 (1.24) | 1.20 (1.07) | 1.42 (1.41) | <0.001 | 1.28 (1.25) | 1.17 (1.10) | 1.40 (1.37) | <0.001 |
| BUN, mg/dL, mean (SD) | 24.31 (17.61) | 22.44 (15.43) | 26.52 (19.65) | <0.001 | 24.13 (17.72) | 21.76 (15.68) | 26.50 (19.27) | <0.001 |
| Uric Acid, mg/dL, mean (SD) | 5.66 (2.86) | 5.36 (2.50) | 5.88 (3.10) | 0.235 | 5.68 (2.68) | 5.58 (2.60) | 5.75 (2.75) | 0.709 |
| TyG index, mean (SD) | 9.16 (0.74) | 8.63 (0.39) | 9.78 (0.53) | <0.001 | 9.20 (0.74) | 8.63 (0.40) | 9.77 (0.52) | <0.001 |
|  | **Events** | | | | | | | |
| LOS ICU, days, mean (SD) | 4.28 (6.08) | 3.73 (5.75) | 4.92 (6.38) | <0.001 | 4.28 (5.78) | 3.71 (5.14) | 4.85 (6.32) | <0.001 |
| LOS Hospital, days, mean (SD) | 8.71 (10.12) | 7.82 (8.52) | 9.76 (11.63) | <0.001 | 8.69 (10.04) | 7.75 (8.34) | 9.63 (11.42) | <0.001 |
| ICU death, n (%) | 258 (8.5) | 111 (6.8) | 147 (10.6) | <0.001 | 216 (8.3) | 80 (6.2) | 136 (10.5) | <0.001 |
| Hospital death, n (%) | 350 (11.6) | 159 (9.7) | 191 (13.7) | 0.001 | 283 (10.9) | 105 (8.1) | 178 (13.7) | <0.001 |
| Follow-up death, n (%) | 1148 (37.9) | 593 (36.2) | 555 (39.9) | 0.041 | 940 (36.2) | 413 (31.8) | 527 (40.5) | <0.001 |

**Abbreviations:** TyG index, triglyceride glucose index; BMI, body mass index; ICU, intensive care unit; SOFA, sequential organ failure assessment; LODS, Logistic organ dysfunction system; OASIS, Oxford acute severity of illness; SIRS, systemic inflammatory response syndrome; APSIII, Acute physiology score III; SAPSII, Simplifed acute physiological score II; CCU, coronary care unit; CSRU, cardiac surgery recovery unit; MICU, medical intensive care unit; SICU, surgical intensive care unit; TSICU, trauma/surgical intensive care unit; HR, heart rate; bmp, beats per minute; SBP, systolic blood pressure; DBP, diastolic blood pressure; SpO_2_, pulse blood oxygen saturation; CHD, coronary heart disease; HF, heart failure; AF, atrial fibrillation; DM, diabetes; COPD, chronic obstructive pulmonary disease; RF, respiratory failure; LD, liver disease; AKI, acute kidney injury; CKD, chronic kidney disease; WBC, white blood cell; RBC, red blood cell; TC, total cholesterol; TG, triglyceride; LDL, low-density lipoprotein; HDL, high-density lipoprotein; HbA1c, hemoglobin A1c; Ucr, urine creatinine; Scr, serum creatinine; BUN, blood urea nitrogen; LOS, length of stay.

**^a^** Lower TyG index (≤9.157); Higher TyG index (>9.157)

**^b^** AKI was defined according to KDIGO guidelines as an increase in serum creatinine (Scr) by ≥0.3 mg/dl (≥26.5 μmol/l) from baseline within 48 hours.
